# Proteomic Analysis of Kidney Preservation Solutions Prior to Renal Transplantation

**DOI:** 10.1371/journal.pone.0168755

**Published:** 2016-12-30

**Authors:** Abdurrahman Coskun, Ahmet Tarik Baykal, Dilek Kazan, Muslum Akgoz, Merve Oztug Senal, Ibrahim Berber, Izzet Titiz, Gokhan Bilsel, Hakan Kilercik, Kubra Karaosmanoglu, Muslum Cicek, Ilknur Yurtsever, Cevat Yazıcı

**Affiliations:** 1 Acibadem University School of Medicine, Department of Medical Biochemistry, Istanbul, Turkey; 2 Marmara University Engineering Faculty, Department of Bioengineering, Istanbul, Turkey; 3 National Metrology Institute, Gebze, Turkey; 4 Acibadem University School of Medicine Department of General Surgery, Istanbul, Turkey; 5 Haydarpasa Numune Research and Training Hospital, Department of General Surgery, Istanbul, Turkey; 6 Yeni Yuzyil University, Gaziosmanpasa Hospital, Department of Anesthesiology, Istanbul, Turkey; 7 Istanbul Medipol University, Regenerative and Restorative Medicine Research Center, Beykoz/Istanbul, Turkey; 8 Erciyes University, School of Medicine, Department of Medical Biochemistry, Kayseri, Turkey; Universidade de Sao Paulo, BRAZIL

## Abstract

One of the main issues in kidney transplantation is the optimal functional preservation of the organ until its transplantation into the appropriate recipient. Despite intensive efforts, the functional preservation period remains limited to hours. During this time, as a result of cellular injury, various proteins, peptides, and other molecules are released by the organ into the preservation medium. In this study, we used proteomic techniques to analyze the protein profiles of preservation solutions in which organs had been preserved prior to their transplantation. Samples were obtained from the preservation solutions of 25 deceased donor kidneys scheduled for transplantation. The protein profiles of the solutions were analyzed using 2D gel electrophoresis/MALDI-TOF and LC-MS/MS. We identified and quantified 206 proteins and peptides belonging to 139 different groups. Of these, 111 proteins groups were belonging to kidney tissues. This study used proteomic techniques to analyze the protein profiles of organ preservation solutions. These findings will contribute to the development of improved preservation solutions to effectively protect organs for transplantation.

## Introduction

The success of transplantation depends not only on the surgical techniques and immunosuppressive agents used, but also on efficient preservation of the organ prior to transplantation. Thus, one of the main issues in transplantation is the optimal functional preservation of the organ until it is transplanted into the appropriate patient.

Without preservation, viability of the organ is limited to a period of 30–60 min after its disconnection from the native circulation [1]. Thereafter, the development of oxygen deficiency, the need for metabolic substrates, and the accumulation of metabolic end products result in progressive organ damage. This is the case in the kidney, in which ischemia and reperfusion injury following transplantation are unavoidable [2]. Even during partial nephrectomy in patients with normal preoperative kidney function, warm ischemia causes a significant deterioration of kidney function, especially after the first 30 min [3]. To efficiently protect organs prior to transplantation, both hypothermia and pharmacological agents are commonly employed. Hypothermia suppresses the metabolic rate during the preservation period; however, when used alone, it does not confer adequate organ protection. Furthermore, it has serious side effects that promote cell injury and play a key role in delayed graft function [4]. This condition is seen in 20–50% of transplanted cadaver kidneys and is a major risk factor influencing both the early and long-term survival of a transplanted organ [4]. Instead, hypothermia is used in combination with pharmacological agents, such as preservation solutions. The latter include the University of Wisconsin, Euro Collins, histidine-tryptophan-ketoglutarate, and Celsior. Yet, despite these and other efforts, the functional preservation period of solid organs is still limited to hours. By contrast, cells such as erythrocytes can be preserved for very long periods of time. Improving the preservation time of solid organs—either by developing new preservation solutions or modifying existing ones—requires a detailed understanding of the pathophysiological changes that occur in the organ during preservation. These changes include cellular injury that leads to the release of enzymes, proteins, and other molecules by the organ into the preservation medium. The proteins and peptides in the preservation solution can be analyzed in detail using proteomic techniques. Therefore, the aim of this study was to use 2D gel electrophoresis/matrix-assisted laser desorption/ionization time-of-flight mass spectrometry (MALDI-TOF) and liquid chromatography-tandem mass spectrometry (LC-MS/MS) to analyze the proteins and peptides that are passed from the kidneys to the preservation solution during the period of organ preservation.

## Material and Methods

### Donor population

Preservation solutions were obtained from those used to preserve 25 deceased donor (14 men and 11 women) kidneys prior to transplantation. We used University of Wisconsin solution to preserve organs prior to transplantation. The median age of donors’ was 48 (range 29–78) years old. All donors were donation after brain death. All kidneys were preserved with static cold storage and the median of cold ischemia time (CIT) was 720 (range 145–1140) minutes. 25 samples were used for two dimensional polyacrylamide gel electrophoresis (2D PAGE) and 18 samples were used for Quadrupole-TOF (Q-TOF) analysis. This study was conducted at the Acibadem University and Marmara University in Istanbul and at the National Metrological Institute, Gebze, Kocaeli, Turkey. The study protocol was approved by the Ethics Committee of Acibadem University and written informed consent from all participants or the next of kin were obtained for the use of the samples in the study.

### Materials

The 2D electrophoresis equipment, i.e., isoelectric focusing (PROTEAN IEF) and Sodium dodecyl sulfate—polyacrylamide gel electrophoresis (protean II XI 2-D cell), the immobilized pH gradient (IPG; BioLyte) strips, mineral oil, glycine, ampholytes, and Ready Prep 2D clean-up kit were obtained from Bio-Rad (Hercules, CA, USA). SDS, tris, urea, thiourea, ammonium bicarbonate, 3-[(3-cholamido propyl) dimethylamonio]-1-propanesulfonate (CHAPS), isopropanol, iodoacetamide, acrylamide, bisacrylamide, the ProteoSilver Plus Silver Stain Kit, glycerol, and α-cyanohydroxycinnamic acid were obtained from Sigma-Aldrich (St. Louis, MO, USA). Tetramethylethylenediamine, dithiothreitol (DTT), ethanol, and methanol were from Merck. (Whitehouse Station, NJ, USA). The 2D Quant kit was purchased from GE Healthcare (Piscataway, NJ, USA). Dimethylated, proteomics grade trypsin from porcine pancreas was obtained from Sigma Aldrich.

### Sample preparation

All preservation solution samples were centrifuged for 15 min at 1500 *g* and stored at −80°C until analysis. The samples were concentrated and prepared for IEF using the Ready Prep 2D clean-up kit. The resulting pellets were resuspended in buffer containing 7 M urea, 2 M thiourea, 4% CHAPS, and 20 mM Tris-HCl, pH 8.8. After centrifugation of the samples at 14,000 *g* for 15 min at 4°C, the supernatant was transferred to a clean microcentrifuge tube, and the protein concentration was determined using the 2D Quant kit according to the manufacturer’s protocol.

### IEF/SDS PAGE and image analysis

The IEF and 2D PAGE analysis were done according to the manufacturer’s (Bio-Rad) protocol. The protein samples (150 μg) were mixed with 315 μl rehydration solution (7 M urea, 2 M thiourea, 4% CHAPS, 1% DTT, 0.2% ampholyte IPG pH 3–10, 5% glycerol, 10% isopropanol) and then incubated at room temperature for 20 min. They were then centrifuged for 5 min at 14,000 *g*, applied to 18-cm pH 3–10 IPG strips, and passively rehydrated overnight at room temperature. The proteins were focused using the following steps: 200 V (hold) for 15 min, 1000 V (gradient) for 3 h, 10000 V (hold) for 3 h, for a total of 55,000 V. Following IEF, the IPG strips were incubated first in 10 mL equilibration buffer (50 mM Tris-HCl, pH 8.8, 6 M urea, 30% glycerol, 2% SDS, 1% DTT, 0.002% bromophenol blue) for 15 min and then in alkylation buffer [50 mM Tris-HCl, pH 8.8, 6 M urea, 30% glycerol, 2% SDS, 4.5% (w/v) iodoacetamide, 0.002% bromophenol blue] for another 15 min. After incubation, the IPG strips were placed on top of the 12.5% SDS polyacrylamide gels and sealed with 1% agarose solution. The running conditions were 16 mA/gel for 30 min followed by 24 mA/gel for 5.5 h or until the dye ran out of the gel. The gels were subsequently stained using the ProteoSilver Plus Silver Stain Kit and imaged. The images were scanned using the HP Scanjet G4050 photo scanner and then analyzed and compared with the control gel image (human plasma) by gel matching. Protein spots that differed from the control were determined manually.

### In-gel protein digestion

The selected protein spots were excised manually from the gels and digested with modified trypsin as follows [5]. Gel pieces destained using the ProteoSilver Plus Stain Kit were washed twice with 50% (v:v) aqueous acetonitrile containing 25 mM ammonium bicarbonate, then once with acetonitrile, and dried in a vacuum concentrator for 20 min. After incubation in 10 mM DTT (in 100 mM ammonium bicarbonate) for 30 min at 56°C and then in 55 mM iodoacetamide solution (in 100 mM ammonium bicarbonate) for 20 min at room temperature in the dark, the proteins were washed with acetonitrile and dried in a vacuum concentrator for 20 min. Proteins in the gel pieces were digested in 30 μl trypsin solution (20 μg trypsin/ml in 0.1 mM HCl, 40 mM ammonium bicarbonate, 9% acetonitrile) overnight at 37°C. The samples were then transferred into clean microcentrifuge tubes and dried to complete dryness in a vacuum concentrator. The peptide mixture was resuspended in 5 μl 0.1% trifluoroacetic acid for MALDI-TOF analysis.

### MALDI-TOF peptide mass fingerprinting analysis

Samples were prepared according to the manufacturer’s protocol [6]. The sample solution was mixed with the CHHA matrix [α-cyano-4-hydroxycinnamic acid saturated in 50:50 (v/v) acetonitrile:trifluoroacetic acid (0.1%) in water] at a 1:1 ratio, deposited onto the ground-steel MALDI MTP 384 target plate (Bruker Daltonics, Bremen, Germany), and allowed to dry. The samples were analyzed using the Autoflex MALDITOF/TOF instrument (Bruker Daltonics) in positive reflectron mode. The instrument was set at the following acquisition parameters: laser; 70%, frequency: 60, mass range acquisition: 500–3500 Da, number of shots: 500, sample rate: 0.5 GS/S, electronic gain: 100 Mv. The spectra were calibrated using an external calibration standard (peptide calibration standard II; Bruker Daltonics). The data were analyzed using the recalibrated peak list generated by Flex Analysis (v. 2.4). The annotated spectra were transferred to BioTools (v. 3.0), with an interface to the MASCOT database search engine (www.matrixscience.com; Matrix Science, London, UK). The database parameters were as follows: SwissProt 2013_02 database; taxonomy: *Homo sapiens*, enzyme: trypsin, global modifications: carbamido methyl cysteine, mass values: MH+ monoisotopic, maximum missed cleavage sites: 1, peptide charge: 1 H+, mass tolerance MS: 100 ppm. Confidence in the peptide identifications was assessed based on the MASCOT sequence assignment score and visual inspection of the molecular mass and the pI values of the selected spots from the gels.

### Q-TOF analysis

In addition to 2D gel electrophoresis/MALDI-TOF, a shotgun proteomic technique was used to obtain the complete protein profile of the preservation solution. Prior to Q-TOF analysis, the solution was subjected to immunodepletion (described below) to remove several of the major proteins that could have masked proteins of interest originally present in the preservation solution at low concentrations.

### Immunodepletion

The Human 14 multiple affinity removal system (Agilent) was used to deplete highly abundant proteins from the preservation solution samples. Briefly, a 4.6× 50mm Hu14 column was equilibrated with buffer A at a flow rate of 0.125 ml/min for 10 min, after which 20 μl preservation solution sample were mixed with 80 μl buffer A and injected onto the column. Isocratic elution of the flow-through fraction with buffer A yielded the protein-depleted preservation solution samples; isocratic elution with buffer B at a flow rate of 1 ml/min yielded the highly bound abundant proteins. All of the samples were lyophilized and dissolved in 200 μl 50 mM ammonium bicarbonate solution and dialyzed overnight against 50 mM ammonium bicarbonate.

### Trypsin digestion

A filter-aided sample preparation method was used to generate the tryptic peptides [7]. Briefly, 50 μg protein solution were incubated at 95°C for 15 min in Universal protein extraction buffer (Expedeon), washed with 6M urea in a 30-kDa cut-off spin column, alkylated with 10 mM iodoacetamide, and trypsinized overnight (1:100trypsin to protein ratio). The resulting tryptic peptides were diluted to 100 μg/μl and transferred to a liquid chromatography vial.

### LC-MS/MS analysis and database search

LC-MS/MS analysis and protein identification were performed following our previously published protocol [8]. Briefly, for each experimental condition, 500 ng tryptic peptides in 5 μl of solution were analyzed using a nano LC-MS/MS system [nano ACQUITY ultra performance liquid chromatography and SYNAPT high definition mass spectrometer with a nano-lock spray ion source; (Waters)]. The columns were equilibrated with 97% mobile phase A (0.1% formic acid in LC-MS grade water (Merck); the column temperature was set to 45°C. The peptides were eluted from the trap column (Symmetry C18 5 μm, 180-μm i.d. × 20 mm, Waters) by gradient elution onto an analytical column (BEH C18, 1.7 μm, 75-μm i.d. × 250 mm, Waters) at a flow rate of 300 nl/min, with a gradient of 5–40% mobile phase B (0.1 formic acid in hyper grade acetonitrile, Merck), over a 90-min period. The MS parameters were set as reported previously [9]. The instrument was run in positive ion V mode, applying the MS and MS/MS functions over 1.5-s intervals with 6-V low-energy and 15- to 40-V high energy collisions. Glu-fibrinopeptide (internal mass calibrant) was infused every 45 s at a flow rate of 300 nl/min. Peptides with *m/z* values of 50–1600 were analyzed. Tandem mass data extraction, charge state deconvolution, and deisotoping were performed using the ProteinLynx Global Server v. 2.5 (Waters) and searched using the IDENTITYE algorithm, with a fragment-ion mass tolerance of 0.025 Da and a parent-ion tolerance of 0.0100 Da against the reviewed *Homo sapiens* protein database from Uniprot. The databank search query was set to a minimum of 3 fragment ion matches per peptide, a minimum of 7 fragment ion matches per protein, a minimum of 1 peptide match per protein, and 1 missed cleavage. The following variable modifications were also set: carbamido methyl-cysteine fixed modification, and acetyl N-TERM, asparagine and glutamine deamidation, and methionine oxidation. Progenesis LC-MS software v. 4.0 (Nonlinear Dynamics) was used to calculate the fold expression changes. Normalization across the sample set was based on the total ion intensities. After chromatographic alignment, normalization, and calculation of the peptide abundances and fold expression changes, an Excel file listing the normalized abundances of all the identified proteins was generated [10].

### Determination of kidney proteins

The database of the Human Kidney and Urine Proteome Project [11] was used to determine kidney-derived proteins and peptides in the immunodepleted samples.

### Statistical analysis

We used Kolmogorov-Smirnov test to evaluate the normality of the data. Correlations between variables were assessed using Spearman correlation analysis. Values of p<0.05 were considered as statistically significant.

## Results

**Fig 1:**
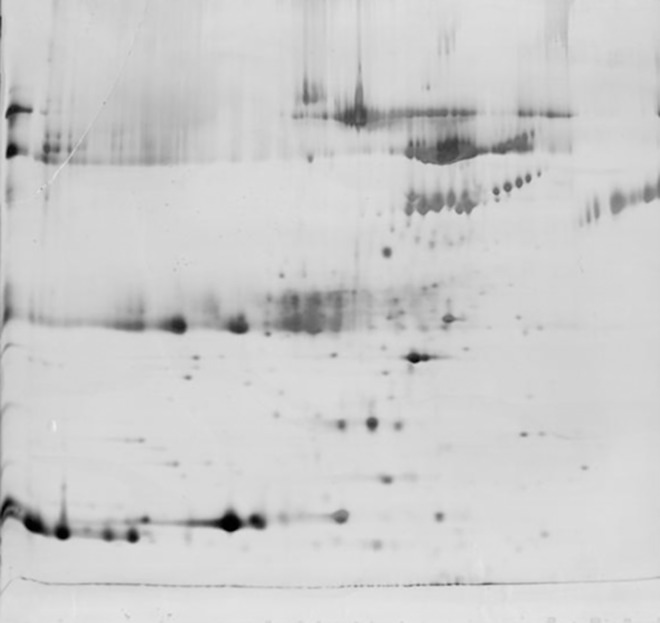
Protein spots of kidney preservation solution identified by 2D gel electrophoresis.

Only a limited number of proteins could be identified using 2D gel electrophoresis (Fig 1), whereas 206 proteins and peptides belonging to 139 different groups were identified and quantified using LC-MS/MS, in the preservation solutions of kidneys prior to transplantation (S1 Table). Within 139 protein groups 111 proteins groups belonged to kidney tissues (Table 1).

**Table 1:** Proteins and peptides belonging to kidney tissues detected from preservation solution using LC-MS/MS (n:18).

**Main Proteins**	**Sub Groups**	**Entry Name (UniProt)**	**Gene Name**	**Accession Number (UniProt)**	**Database (G,M)**
**Actin**					
	Actin cytoplasmic 1	ACTB	ACTB	P60709	G,M
	Actin cytoplasmic 2	ACTG	ACTG1	P63261	G
	Actin alpha cardiac muscle 1	ACTC	ACTC1	P68032	G,M
	Beta actin like protein 2	ACTBL	ACTBL2	Q562R1	M
	Putative beta actin like protein 3	ACTBM	POTEKP	Q9BYX7	M
**Actinin**	Alpha actinin 4	ACTN4	ACTN4	O43707	G,M
**Transgelin**					
	Transgelin	TAGL	TAGLN	Q01995	G,M
	Transgelin 2	TAGL2	TAGLN2	P37802	G,M
**Vitamin D Binding Protein**	Vitamin D binding protein	VTDB, D6RF35	GC	P02774	M
**Leucine rich alpha 2 glycoprotein**	Leucine rich alpha 2 glycoprotein	A2GL	LRG1	P02750	M
**Lumican**	Lumican	LUM	LUM	P51884	G,M
**Collagen**					
	Collagen alpha 3 VI chain	CO6A3	COL6A3	P12111	G,M
	Collagen alpha 2 VI chain	CO6A2	COL6A2	P12110	G,M
	Collagen alpha 1 VI chain	CO6A1	COL6A1	P12109	G,M
	Collagen alpha 1 XVIII chain	COIA1	COL18A1	P39060	G,M
**Vimentin**	Vimentin	VIME	VIM	P08670	G,M
**Heat Shock Protein**					
	Heat shock cognate 71 kDa protein	HSP7C	HSPA8	P11142	G,M
	Heat shock 70 kDa protein 1A and 1B	HS71A and HS71B	HSPA1A and HSPA1B	P0DMV8 and P0DMV9 (old number P08107)	G,M
	Heat shock protein beta 1	HSPB1	HSPB1	P04792	G,M
	Heat shock 70 kDa protein 6	HSP76	HSPA6	P17066	G
	Heat shock protein HSP 90 beta	HS90B	HSP90AB1	P08238	M
	60 kDa heat shock protein mithocondrial	CH60	HSPD1	P10809	G,M
**Protein 14 3 3**					
	14 3 3 protein zeta delta	1433Z	YWHAZ	P63104	G,M
	14 3 3 protein epsilon	1433E	YWHAE	P62258	G,M
	14 3 3 protein gamma	1433G	YWHAG	P61981	G,M
	14 3 3 protein beta alpha	1433B	YWHAB	P31946	G,M
	14 3 3 protein eta	1433F	YWHAH	Q04917	G,M
**Gelsolin**	Isoform 2 of Gelsolin	GELS	GSN	P06396	G,M
**Cofilin**	Cofilin 1	COF1	CFL1	P23528	G,M
**Vitronectin**	Vitronectin	VTNC	VTN	P04004	G,M
**Tubulin**					
	Tubulin beta chain	TBB5	TUBB	P07437	G,M
	Tubulin alpha 1B chain	TBA1B	TUBA1B	P68363	M
**Clusterin**	Clusterin	CLUS	CLU	P10909	G,M
**Annexin**					
	Annexin A5	ANXA5	ANXA5	P08758	G,M
	Annexin A1	ANXA1	ANXA1	P04083	G,M
**Tropomyosin**					
	Isoform 2 of Tropomyosin beta chain	TPM2	TPM2	P07951-2	G,M
	Tropomyosin beta chain	TPM2	TPM2	P07951	G,M
**Prelamin**	Prelamin A C	LMNA	LMNA	P02545	G,M
**Myosin**					
	Myosin 9	MYH9	MYH9	P35579	G,M
	Myosin light polypeptide 6	MYL6	MYL6	P60660	G,M
	Isoform 2 of Myosin 11	MYH11	MYH11	P35749-2	G,M
**Amyloid P**	Serum amyloid P component	SAMP	APCS	P02743	G,M
**Profilin**	Profilin 1	PROF1	PFN1	P07737	G,M
**Alpha Crystallin**	Alpha crystallin B chain	CRYAB	CRYAB	P02511	G,M
**Cathepsin D**	Cathepsin D	CATD	CTSD	P07339	G,M
**Galectin**	Galectin 1	LEG1	LGALS1	P09382	G,M
**Keratin**					
	Keratin type II cytoskeletal 8	K2C8	KRT8	P05787	M
	Keratin type I cytoskeletal 18	K1C18	KRT18	P05783	M
	Keratin type I cytoskeletal 19	K1C19	KRT19	P08727	M
**Thymosin**	Thymosin beta 4	TYB4	TMSB4X	P62328	M
**Filamin**	Filamin A	FLNA	FLNA	P21333	G,M
**Rab GDP Dissociation Inhibitor**	Rab GDP dissociation inhibitor beta	GDIB	GDI2	P50395	G,M
**Protein DJ**	Protein DJ 1	PARK7	PARK7	Q99497	G,M
**Transforming Growth Factor**	Transforming growth factor-beta-induced protein ig-h3	BGH3	TGFBI	Q15582	G,M
**Rho GDP Dissociation Inhibitor**	Rho GDP Dissociation inhibitor 1	GDIR	ARHGDIA	P52565	G,M
**LIM Domains**	Four and a half LIM domains protein 1	FHL1	FHL1	Q13642	G,M
**Calmodulin**	Calmodulin	CALM	CALM1	P62158	G
**Beta 2 Microglobulin**	Beta 2 microglobulin	B2MG	B2M	P61769	G
**Microfibril Accociated Glycoprotein**	Microfibril associated glycoprotein 4	MFAP4	MFAP4	P55083	G,M
**Vinculin**	Vinculin	VINC	VCL	P18206	G,M
**Basement Membrane Specific Heparan Sulfate Proteoglycan Core Protein**	Basement membrane specific heparin sulfate proteoglycan core protein	PGBM	HSPG2	P98160	G,M
**Lysozyme C**	Lysozyme C	LYSC	LYZ	P61626	G,M
**Mimecan**	Mimecan	MIME	OGN	P20774	G,M
**Serotransferrin**	Serotransferrin	TRFE	TF	P02787	G,M
**Caldesmon**	Caldesmon	CALD1	CALD1	Q05682	G,M
**Talin**	Talin 1	TLN1	TLN1	Q9Y490	G,M
**Myomegalin**	Myomegalin	MYOME	PDE4DIP	Q5VU43	M
**Alpha 1 anti-chymotrypsin**	Alpha 1 anti-chymotrypsin	AACT	SERPINA3	P01011	G,M
**Ceruloplasmin**	Ceruloplasmin	CERU	CP	P00450	M
**Complement**					
	Complement C4 A	CO4A	C4A	P0C0L4	G,M
	Complement C4 B	CO4B	C4B	P0C0L5	G,M
	Complement factor B	CFAB	CFB	P00751	M
	Complement factor I	CFAI	CFI	P05156	M
	Complement C3	CO3	C3	P01024	G,M
	Complement component C9	CO9	C9	P02748	G,M
**Trypsin**					
	Isoform 2 of Inter alpha trypsin inhibitor heavy chain H4	ITIH4	ITIH4	Q14624	M
	Alpha 1 antitrypsin	A1AT	SERPINA1	P01009	G,M
	Inter alpha trypsin inhibitor heavy chain H1	ITIH1	ITIH1	P19827	M
**Heomopexin**	Hemopexin	HEMO	HPX	P02790	M
**Enolase**	Alpha enolase	ENOA	ENO1	P06733	G,M
**Carbonic Anhydrase**					
	Carbonic anhydrase 1	CAH1	CA1	P00915	G,M
	Carbonic anhydrase 2	CAH2	CA2	P00918	G,M
**Angiotensinogen**	Angiotensinogen	ANGT	AGT	P01019	G,M
**Fatty Acid Binding Protein Adipocyte**	Fatty acid binding protein adipocyte	FABP4	FABP4	P15090	G,M
**Alcohol dehydrogenase**	Alcohol dehydrogenase 1B	ADH1B	ADH1B	P00325	M
**Alpha 1B glycoprotein**	Alpha 1B glycoprotein	A1BG	A1BG	P04217	M
**Triosephosphate Isomerase**	Triosephosphate isomerase	TPIS	TPI1	P60174	G,M
**Glyceraldehyde 3 Phosphate Dehydrogenase**	Glyceraldehyde 3 phospate dehydrogenase	G3P	GAPDH	P04406	G,M
**Fructose 1 6 Bisphosphatase**	Fructose 1 6 bisphosphatase 1	F16P1	FBP1	P09467	G,M
**Fructose Bisphosphate Aldolase**					
	Fructose bisphosphate aldolase A	ALDOA	ALDOA	P04075	G,M
	Fructose bisphosphate aldolase B	ALDOB	ALDOB	P05062	G,M
	Fructose bisphosphate aldolase C	ALDOC	ALDOC	P09972	G,M
**Peptidyl Prolyl cis trans Isomerase**	Peptidyl prolyl cis trans isomerase A	PPIA	PPIA	P62937	G,M
**Peroxiredoxin**					
	Peroxiredoxin 6	PRDX6	PRDX6	P30041	G,M
	Peroxiredoxin 2	PRDX2	PRDX2	P32119	G,M
	Peroxiredoxin 1	PRDX1	PRDX1	Q06830	G,M
**Phosphatidyl ethanolamine binding protein**	Phosphatidyl ethanolamine binding protein	PEBP1	PEBP1	P30086	M
**Alpha 2 HS glycoprotein**	Alpha 2 HS glycoprotein	FETUA	AHSG	P02765	M
**Lactate Dehydrogenase**					
	L-lactate dehydrogenase A chain	LDHA	LDHA	P00338	G,M
	L-lactate dehydrogenase B chain	LDHB	LDHB	P07195	G,M
**Glycerol 3 Phosphate Dehydrogenase**	Glycerol 3 phosphate dehydrogenase	GPDA	GPD1	P21695	G,M
**Phosphoglycerate Mutase**	Phosphoglycerate mutase 1	PGAM1	PGAM1	P18669	G,M
**Phosphoglycerate Kinase**	Phosphoglycerate kinase 1	PGK1	PGK1	P00558	G,M
**Glutathione S Transferase**					
	Glutathione S transferase A1	GSTA1	GSTA1	P08263	G
	Glutathione S transferase P	GSTP1	GSTP1	P09211	G,M
**Histone**					
	Histone H2A type 1A	H2A1A	HIST1H2AA	Q96QV6	G
	Histone H2A type 1D	H2A1D	HIST1H2AD	P20671	G
	Histone H1 4	H14	HIST1H1E	P10412	G,M
	Histone H3 3	H33	H3F3A	P84243	G,M
	Histone H4	H4	HIST1H4A	P62805	G,M
	Histone H2B type 1B	H2B1B	HIST1H2BB	P33778	G
**Antithrombin III**	Antithrombin III	ANT3	SERPINC1	P01008	G,M
**Adipose most abundant gene transcript 2 protein**	Adipose most abundant gene transcript 2 protein	ADIRF	ADIRF	Q15847	M
**Selenium Binding Protein**	Selenium binding protein 1	SBP1	SELENBP1	Q13228	G,M
**ATP Synthase**	ATP synthase subunit beta mitocondrial	ATPB	ATP5B	P06576	G,M
**Aldo Keto Reductase**	Aldo keto reductase	AK1C1	AKR1C1	Q04828	M
**Apolipoprotein A**					
	Apolipoprotein A I	APOA1	APOA1	P02647	G,M
	Apolipoprotein A II	APOA2	APOA2	P02652	G
	Apolipoprotein A IV	APOA4	APOA4	P06727	G,M
**Pyruvate Kinase**	Pyruvat kinase isoenzymes M1 M2	KPYM	PKM	P14618	G,M
**Glucose 6 Phosphate Isomerase**	Glucose 6 phosphate isomerase	G6PI	GPI	P06744	G,M
**LMW of Kininogen**	Isoform LMW of Kininogen 1	KNG1	KNG1	P01042-2	G,M
**Retinal Dehydrogenase**	Retinal dehydrogenase 1	AL1A1	ALDH1A1	P00352	G,M
**Catalase**	Catalase	CATA	CAT	P04040	G,M
**Extracellular Superoxide Dismutase**	Extracellular superoxide dismutase Cu Zn	SODE	SOD3	P08294	G,M
**Isocitrate Dehydrogenase**	Isocitrate dehydrogenase NADP cytoplasmic	IDHC	IDH1	O75874	G,M
**Acetyl CoA Acetyl Transferase**	Acetyl CoA acetyl transferase mitocondrial	THIL	ACAT1	P24752	G,M
**3 Ketoacyl CoA Thiolase**	3 ketoacyl CoA thiolase mitocondrial	THIM	ACAA2	P42765	G,M
**Thyroxine binding globulin**	Thyroxine binding globulin	THBG	SERPINA7	P05543	M
**Phospho Glucomutase**	Phospho glucomutase 1	PGM1	PGM1	P36871	G,M
**Protein S100 A6**	Protein S100 A6	S10A6	S100A6	P06703	M
**Flavin Reductase**	Flavin reductase NADPH	BLVRB	BLVRB	P30043	G,M
**Elongation Factor**	Elongation factor 1 alpha 1	EF1A1	EEF1A1	P68104	G,M
**Malate Dehydrogenase**	Malate dehydrogenase cytoplasmic	MDHC	MDH1	P40925	G,M
**Transketolase**	Transketolase	TKT	TKT	P29401	G,M
**Inorganic Pyrophosphatase**	Inorganic pyrophosphatase	IPYR	PPA1	Q15181	G,M
**Betaine Homocysteine S Methyl Transferase**	Betaine homocysteine S Methyl Transferase 1	BHMT1	BHMT	Q93088	G,M
**Cytosolic Nonspecific Dipeptidase**	Cytosolic nonspecific dipeptidase	CNDP2	CNDP2	Q96KP4	G,M
**Aminoacylase**	Aminoacylase 1	ACY1	ACY1	Q03154	G,M
**Fumaryl acetoacetase**	Fumaryl acetoacetase	FAAA	FAH	P16930	G,M
**Aldose Reductase**	Aldose reductase	ALDR	AKR1B1	P15121	G,M
**Metallothionein 1E**	Metallothionein 1E	MT1E	MT1E	P04732	M
**Hydroxyacyl Coenzyme A Dehydrogenase**	Hydroxyacyl coenzyme A dehydrogenase mitocondrial	HCDH	HADH	Q16836	M
**Splicing factor 3B subunit 3**	Splicing factor 3B subunit 3	SF3B3	SF3B3	Q15393	M
**Adenylyl cyclase associated protein**	Adenylyl cyclase associated protein	CAP1	CAP1	Q01518	G,M
**Alpha 2 Macroglobulin**	alpha 2 macroglobulin	A2MG	A2M	P01023	G,M
**Ester Hydrolase**	Ester hydrolase C11orf54	CK054	C11orf54	Q9H0W9	M
**Ferritin**	Ferritin heavy chain	FRIH	FTH1	P02794	G
**NADP dependent malic enzyme**	NADP dependent malic enzyme	MAOX	ME1	P48163	M
**6 Phospho Gluconate Dehydrogenase Decarboxylating**	6 phospho gluconate dehydrogenase decarboxylating	6PGD	PGD	P52209	G,M
**Plasma protease C1 inhibitor**	Plasma protease C1 inhibitor	ICI	SERPING1	P05155	M
**Delta aminolevulinic acid dehydratase**	Delta aminolevulinic acid dehydratase	HEM2	ALAD	P13716	G,M
**Cysteine and Glycine Rich Protein**	Cystein and glycine rich protein 1	CSRP1	CSRP1	P21291	G,M
**Polymerase I and Transcript Release Factor**	Polymerase I and transcript release factor	PTRF	PTRF	Q6NZI2	G,M
**Carbonyl Reductase**	Carbonyl reductase NADPH1	CBR1	CBR1	P16152	G,M
**Haptoglobulin**	Haptoglobulin	HPT	HP	P00738	G,M

G: Glomerulus database; M: Medulla database (http://www.hkupp.org/)

The hematological and biochemical test results of patients’ (n: 18) followed up for one year are given as supplemental data (S2 Table). The median and interquartile range of recipients’ serum BUN and creatinine levels were 8.9 mmol/L (7.3–10.9) and 120.2 μmol/L (102.5–156.5) respectively. As shown in Table 2, there were significant correlations between the levels of proteins (based on their ion intensities) of preservation solutions and donors’ age (23 proteins), CIT (5 proteins), recipients’ serum BUN (12 proteins) and creatinine (7 proteins) levels. Therefore, the levels of these proteins in preservation solution can potentially be used as a reporter of marginality of kidney prior to transplantation. Additionally, the levels of these proteins can be used also to evaluate the viability of organs prior to transplantation.

**Table 2:** List of proteins and peptides that were isolated from kidney preservation solution which showed a statistically significant correlation with donors’ age (DA), cold ischemia time (CIT), creatinine (Cr) and blood urea nitrogen (BUN).

**Protein and peptide**	**Significant Correlation**	**Correlation coefficient**	**P**
**Actin cytoplasmic 1**	DA	0.551	0.022
**Adenylyl cyclase associated protein 1**	DA	0.506	0.038
**Aldo keto reductase family 1 member C1**	DA	0.676	0.030
**Aldose reductase**	DA	0.584	0.014
**Alpha 2 macroglobulin**	DA*, Cr^†^,	0.648*,0.512^†^	0.005*, 0.030^†^
**Aminoacylase 1**	DA	0.506	0.038
**Antithrombin III**	DA*, Cr^†^, BUN^‡^	0.506*, 0.533^†^, 0.610^‡^	0.038*, 0.023^†^, 0.007^‡^
**ATP synthase subunit beta mitochondrial**	CIT	0.518	0.028
**Basement membrane specific heparan sulfate proteoglycan core protein (Perlecan)**	Cr^†^, BUN^‡^	0.518^†^, 0.599^‡^	0.028^†^, 0.009^‡^
**Cathepsin D**	DA	0.634	0.006
**Ceruloplasmin**	BUN	0.609	0.007
**Collagen alpha 1 VI chain**	DA*, BUN^‡^	0.555*, 0.488^‡^	0.021*,0.040^‡^
**Complement C2**	BUN	0.580	0.012
**Cytosolic non specific dipeptidase**	BUN	0.475	0.046
**Isoform 2 of E3 ubiquitin protein ligase RNF135**	BUN	0.563	0.011
**Fatty acid binding protein adipocyte**	DA	0.622	0.008
**Fructose 1 6 bisphosphatase 1**	DA	0.487	0.047
**Fumaryl acetoacetase**	BUN	0.471	0.049
**Glyceraldehyde 3 phosphate dehydrogenase**	DA	0.519	0.033
**Heat shock protein HSP 90 beta**	CIT	0.517	0.028
**Heat shock 70 kDa protein 6**	DA	0.519	0.033
**Hemopexin**	BUN	0.471	0.049
**Histone H2A type 1 A**	Cr^†^, BUN^‡^	0.656^†^, 0.496^‡^	0.03^†^, 0.036^‡^
**Hydroxyacyl coenzyme A dehydrogenase mitochondrial**	CIT	0.487	0.40
**Keratin type II cytoskeletal 8**	Cr^†^	0.481	0.043
**3 ketoacyl CoA thiolase mitochondrial**	DA	0.693	0.002
**Malate dehydrogenase cytoplasmic**	DA	0.512	0.036
**Microfibril associated glycoprotein 4**	DA*, Cr^†^	0.634*, 0.502^†^	0.006*, 0.034^†^
**Isoform 2 of Myosin 11**	DA	0.564	0.018
**Myosin 9**	DA	0.497	0.042
**Myosin light polypeptide 6**	DA	0.638	0.006
**Peptidylprolylcis trans isomerase A**	DA	0.485	0.048
**Phosphatidyl ethanolamine binding protein 1**	DA	0.502	0.040
**Isoform 2 of Polyamine modulated factor 1**	Cr	0.483	0.042
**Profilin 1**	DA*, BUN^‡^	0.579*, 0.602^‡^	0.015*, 0.008^‡^
**Retinal dehydrogenase 1**	BUN	0.496	0.036
**Rho GDP dissociation inhibitor 1**	CIT	0.476	0.046
**Talin 1**	CIT	0.437	0.022
**Thyroxine binding globulin**	DA	0.532	0.028

*, Donors’ age

^†^, Creatinine

^‡^, Blood urea nitrogen (BUN)

Functional classification of the identified proteins was done with PANTHER overrepresentation test (http://www.pantherdb.org/). The test was run for the classifications based on pathways, molecular function, biological processes, cellular compartment, and protein class (Table 3). Based on the test the identified proteins exhibit overrepresentation of glycolysis related proteins (*p* = 9.85E-13) (Table 3A). The top 3 group of the identified proteins have molecular functions related to structural constituent of cytoskeleton (*p* = 1.09E-10), serine-type endopeptidase inhibitor activity (*p* = 7.73E-09), and peptidase inhibitor activity (*p* = 1.70E-08) (Table 3B). A large number of the identified proteins play a role in biological processes like cellular component organization or biogenesis (*p* = 4.47E-16) and cellular component morphogenesis (*p* = 4.57E-14) (Table 3C). PANTHER GO-Slim database shows that there is a overrepresentation of proteins from cellular compartments of actin cytoskeleton (*p* = 1.08E-07), and cytoskeleton (*p* = 6.29E-07) (Table 3D). The major overpresentation with the highest fold enrichment in terms of protein class was found to be histone (*p* = 1.82E-19), serine protease inhibitor (*p* = 8.04E-09) and actin family cytoskeletal protein (*p* = 1.89E-08) (Table 3E).

**Table 3:** PANTHER Overrepresentation test (release 20160715) with annotation version 11.1. Classification of identified proteins based on A) Pathways, B) Molecular Function, C) Biological Process, D) Cellular Component and E) Protein Class.

	**Homo sapiens (REF)**	**Identified Proteins**			
**A. PANTHER Pathways**	#	#	Expected	Fold Enrichment	P
**Glycolysis**	22	10	0.18	55.75	9.85E-13
**Pentose phosphate pathway**	8	3	0.07	45.99	6.84E-03
**Fructose galactose metabolism**	12	3	0.1	30.66	2.26E-02
**Cytoskeletal regulation by Rho GTPase**	83	8	0.68	11.82	8.24E-05
**Parkinson disease**	100	9	0.82	11.04	2.80E-05
**Integrin signalling pathway**	192	13	1.57	8.3	1.41E-06
**B. PANTHER GO-Slim Molecular Function**					
**Antioxidant activity**	30	5	0.24	20.44	9.88E-04
**Peroxidase activity**	27	4	0.22	18.17	1.39E-02
**Serine-type endopeptidase inhibitor activity**	123	13	1	12.96	7.73E-09
**Peptidase inhibitor activity**	161	14	1.31	10.66	1.70E-08
**Structural constituent of cytoskeleton**	594	27	4.84	5.57	1.09E-10
**Actin binding**	177	8	1.44	5.54	2.05E-02
**Peptidase activity**	525	18	4.28	4.2	6.54E-05
**Oxidoreductase activity**	629	21	5.13	4.09	1.01E-05
**Structural molecule activity**	927	27	7.56	3.57	1.87E-06
**Catalytic activity**	5090	65	41.5	1.57	7.89E-03
**C. PANTHER GO-Slim Biological Process**					
**Glycolysis**	34	8	0.28	28.86	1.40E-07
**Pentose-phosphate shunt**	13	3	0.11	28.3	4.35E-02
**Gluconeogenesis**	22	4	0.18	22.3	8.73E-03
**Tricarboxylic acid cycle**	27	4	0.22	18.17	1.92E-02
**Chromatin organization**	263	20	2.14	9.33	2.16E-11
**Cytokinesis**	113	7	0.92	7.6	1.10E-02
**Monosaccharide metabolic process**	120	7	0.98	7.15	1.60E-02
**Generation of precursor metabolites and energy**	224	12	1.83	6.57	9.74E-05
**Muscle contraction**	154	8	1.26	6.37	1.08E-02
**Cellular component morphogenesis**	545	18	4.44	4.05	1.54E-04
**Cellular component organization or biogenesis**	1724	54	14.06	3.84	4.47E-16
**Cellular component organization**	1584	49	12.92	3.79	4.57E-14
**Organelle organization**	752	21	6.13	3.42	2.53E-04
**D. PANTHER GO-Slim Cellular Component**					
**Intermediate filament cytoskeleton**	71	5	0.58	8.64	1.92E-02
**Actin cytoskeleton**	240	15	1.96	7.67	1.08E-07
**Cytoskeleton**	624	22	5.09	4.32	6.29E-07
**Extracellular space**	530	14	4.32	3.24	7.72E-03
**Extracellular region**	832	18	6.78	2.65	1.04E-02
**Membrane**	2083	3	16.98	< 0.2	1.32E-03
**E. PANTHER Protein Class**					
**Complement component**	14	5	0.11	43.8	2.79E-05
**Histone**	99	20	0.81	24.78	1.82E-19
**Peroxidase**	23	4	0.19	21.33	8.64E-03
**Actin and actin related protein**	30	5	0.24	20.44	1.14E-03
**Serine protease inhibitor**	122	13	0.99	13.07	8.04E-09
**Protease inhibitor**	241	16	1.97	8.14	4.29E-08
**Lyase**	151	8	1.23	6.5	7.88E-03
**Actin family cytoskeletal protein**	389	20	3.17	6.31	1.89E-08
**Chaperone**	183	9	1.49	6.03	4.61E-03
**Dehydrogenase**	253	12	2.06	5.82	2.86E-04
**Oxidoreductase**	597	22	4.87	4.52	9.60E-07
**Cytoskeletal protein**	778	27	6.34	4.26	5.23E-08
**DNA binding protein**	824	22	6.72	3.27	2.38E-04

## Discussion

The contents of a pre-transplantation organ preservation solution provide a “liquid biopsy” of the transplanted organs and therefore carry valuable information about their survival and functionality. We isolated 111 kidney-derived proteins and peptide groups from the pre-transplantation preservation solution. Further analysis of the proteins showed that they originated from both extracellular and intracellular regions of the kidney tissue.

Many of these proteins and peptides were derived from the cytoskeleton and extracellular structures that form the tissue integrity. A group of these proteins showed significant correlations with DA, CIT, recipients’ BUN and creatinine levels (Table 2) as summarized below. Therefore, we propose that these proteins might serve as potential biomarkers for renal injury prior to transplantation.

Ischemia triggers a complex series of biochemical reactions that primarily effect the cytoskeleton [12], including the loss of cellular polarity and cytoskeletal reorganization. Thus, our finding of structural proteins in the kidney preservation solution was probably not a coincidence. For example, the expression and redistribution of microtubule cytoskeleton components, was observed to be induced after renal ischemia–reperfusion injury suggesting the participation of these proteins in an adaptive response to cellular lesions [13]. Likewise, another member of the cellular cytoskeleton, the actin filament network gets disrupted in ischemic kidney leading to the loss of tubule-cell polarity and redistribution of the basolateral membrane [14]. Consequently, the tight junction barrier becomes permeable [15], and cell-cell and cell-substrate adhesions are lost, resulting in the detachment of tubule cells from the basement membrane [12,16]. Alterations in the renal cell cytoskeleton related to ischemia may also induce apoptosis [12]. Indeed, chemically induced apoptosis is frequently preceded by the disorganization of the *F*-actin cytoskeleton [12,17]. Not surprisingly, the level of profilin, a major actin monomer interacting protein, was also correlated with the donors’ age and recipients’ serum BUN levels. Profilins were previously shown to be up-regulated in mesangial proliferative glomerulonephritis [18].

We found a positive significant correlation between perlecan and recipients’ serum BUN and creatinine levels. Perlecan accumulates during glomerular and vascular tissue remodeling that characterizes chronic transplant dysfunction (CTD) in rats [19].

In our study, we found a positive correlation between CIT and talin 1 which is pivotal for the activation of integrins and links them directly to the actin cytoskeleton. These interactions are important for attaching podocytes -specialized actin-rich epithelial cells -to the glomerular basement membrane [20]. Mice lacking talin1 specifically in their podocytes display severe proteinuria, foot process effacement, and kidney failure [20]. All these findings suggest that talin 1 could indeed be a strong candidate to evaluate the viability of the kidney prior to transplantation. We found a positive significant correlation between keratin type II cytoskeletal 8 and recipients’ serum creatinine levels (Table 2). Keratin is expressed by renal tubular epithelia cells and it was implicated with damaged tubular epithelial cell [21].

In our study we isolated myosin 6, 9 and 11 in preservation solution and found positive correlation with donors’ age. Several members of myosin superfamily are synthesized in podocytes. They form a network and their interaction with the actin cytoskeleton is crucial for the regulation of podocyte structure and function [22]. Myosins also have tubular functions and it was shown that myosin 9a-deficient mice develop proteinuria [23]. Another protein isolated in our work is collagen VI, which is produced by endothelial cells. This protein was significantly correlated with DA and recipients’ BUN levels. It has been shown that, antibodies recognizing collagen α1(VI)/α5(IV) play a crucial role in the pathogenesis of transplant glomerulopathy in rats[24]. Also, we isolated microfibril associated glycoprotein 4 from preservation solution and the level of this protein was significantly correlated with DA and recipients’ creatinine level. To the best of our knowledge this glycoprotein has previously not been linked to renal injury.

A second important group of proteins that we isolated from the preservation solution and found positive correlation with donors’ age, CIT, recipients’ BUN and creatinine levels comprised protective and metabolically active proteins (Table 2). These are also candidate biomarkers of kidney injury and summarized below briefly.

Aminoacylase 1 is a zinc-binding cytoplasmic protein which was shown to have its highest level of activity and expression in the kidney [25]. In renal transplant patients, serum aminoacylase-1 level has been used as a biomarker to evaluate the long-term outcome with delayed graft function [26]. Fructose-1,6-bisphosphatase (FBP-1) is one of the key enzymes of gluconeogenesis in the cytosol and mainly found in proximal renal tubules. Urine FBP-1 [27,28] levels were found to be associated with proximal tubular damage. Similarly, alpha 2 macroglobulin [29], aldose reductase [30], antithrombin III [31], ceruloplasmin [32], complements [33], hemopexin [34], histone [35] and adipocyte fatty acid binding protein [36] have all been implicated to associate with renal injury.

Renal cells demand high levels of energy due to their active energy-dependent functions such as reabsorption, secretion and filtration of many substances. Therefore, mitochondrial homeostasis is crucial for normal renal function. In our study we found significant correlation between the CIT and the levels of mitochondrial hydroxyl acyl coenzyme A dehydrogenase and ATP synthase subunit beta which suggests an increase in mitochondrial disruption with CIT. Whitaker et al. have recently shown increased urinary ATP synthase subunit beta in mice with renal injury and concluded that urinary ATP synthase subunit beta may be a novel and sensitive biomarker of renal mitochondrial dysfunction [37].

Other potential biomarkers of renal injury that were identified in our study based on their correlation with donors’ age, CIT, recipients’ serum BUN or creatinine levels are glyceraldehyde 3-phosphate dehydrogenase, malate dehydrogenase, peptidyl prolyl cis trans isomerase, mitochondrial 3 keto acyl CoA thiolase, adenylyl cyclase associated protein, aldo keto reductase, phosphatidyl ethanolamine binding protein, thyroxine binding globulin, cytosolic nonspecific dipeptidase, Isoform 2 of E3 ubiquitin protein ligase RNF135, fumaryl acetoacetase, isoform 2 of Polyamine modulated factor 1, retinal dehydrogenase 1 and Rho GDP dissociation inhibitor 1. Interestingly, these proteins were previously not been associated with renal injury. Therefore, it would be important to validate their potential link to renal disease in an independent experimental workflow.

During hypothermia, the metabolic rate of the kidney is depressed significantly. However, during the preservation period, some metabolic pathways are activated to protect renal tissues. In previous work, we showed that HSP levels are elevated in liver tissues placed in preservation solution prior to transplantation [38]. In the present study, several HSPs (Table 1) were detected in the preservation solution of the pre-transplanted kidney. Furthermore HSP90 beta and HSP70 protein 6 were correlated with CIT and donors’ age respectively (Table 2). HSPs are abundant intracellular proteins with functions including the regulation of protein complex formation, protein trafficking, targeting of misfolded proteins for proteasomal degradation, prevention of unfolded protein aggregation, refolding of denatured proteins, mitochondrial protein folding and assembly, and inhibition of apoptosis [39]. In the kidney, HSPs help maintain and restore normal cellular function following ischemia-reperfusion injury [40].

### Limitations of the study

This study has some limitations that should be noted. During surgery, the blood is washed out of the kidney, and the organ’s vascular system is filled with preservation solution. However, the interstitial edema that occurs during the flushing-out period increases compression of the renal capillaries, which prevents complete removal of blood from the vascular system. We therefore could not confirm that the washing procedure was sufficiently thorough to remove blood from the kidney completely, and the blood may thus have remained in the preservation solution. To avoid artifacts related to the presence of blood and its constituents, the preservation solution samples were subjected to immunodepletion, which removed the most of these components. In future studies of deceased donor kidney transplantation, both immunodepletion and the use of a kidney proteomic database as a reference will contribute to avoiding study artifacts.

### Conclusion

Taken together, in the present study we have shown that various extracellular proteins are released from the kidney to the preservation solution suggesting that the homeostasis of the extracellular matrix might be deteriorated during the preservation period. The dysregulation of extracellular matrix leads to interstitial fibrosis and cathepsin D may promote this process [41]. Currently, many transplant centers are accepting organs from older donors, even those with co-morbidities, due to the shortage of young healthy donors. Organs harvested from marginal donors are more prone to delayed graft function and therefore its adverse consequences. The proteins that show a positive correlation with donors’ age, CIT, recipients’ serum BUN and creatinine levels can be useful markers to assess the marginality and viability of organs prior to transplantation. Further clinical studies are required to evaluate the use of these proteins as potential biomarkers prior to their implementation in clinical practice. Moreover, the development of new preservation solutions which are capable of preserving organs effectively prior to transplantation remains a critical issue in clinical transplantation practice. The protein profile detected in the present study can be used to design new pharmacological agents that stabilize the cytoskeleton of renal cells and maintain the integrity of the kidney’s extracellular tissues. These will improve both organ preservation and transplant success.

## Supporting Information

S1 Table Main Dataset. Proteins and peptides isolated from preservation solution of kidneys prior to transplantation
(XLSX)

S2 Table Receipents test results (Biochemistry and hematology). Main biochemical and hematological test results of recipients during follow up period.
(XLSX)

## Abbreviations

CHAPS: 3-[(3-cholamido propyl) dimethylamonio]-1- propanesulfonate
CIT: Cold ischemia time
DTT: Dithiothreitol
HSP: Heat shock protein
IEF: Isoelectric focusing
IPG: Immobilized pH gradient
LC-MS/MS: Liquid chromatography-tandem mass spectrometry
MALDI-TOF: Matrix-assisted laser desorption/ionization time-of-flight
NADPH: Reduced nicotinamide adenine dinucleotide phosphate
Q-TOF: Quadrupole- time-of-flight
TGF: Transforming growth factor
2D PAGE: Two dimensional polyacrylamide gel electrophoresis
